# “Advances in Preterm Delivery”—How Can We Advance Further?

**DOI:** 10.3390/jcm11123436

**Published:** 2022-06-15

**Authors:** Tamar Wainstock, Eyal Sheiner

**Affiliations:** 1 Department of Public Health, Faculty of Health Sciences, Ben-Gurion University of the Negev, Beer-Sheva 8410501, Israel; wainstoc@bgu.ac.il; 2 Department of Obstetrics and Gynecology, Soroka University Medical Center, Ben-Gurion University of the Negev, Beer-Sheva 8410501, Israel

Preterm delivery (PTD: <37 gestational weeks) complicates 5–13% of deliveries worldwide [1], and is a leading cause of perinatal and childhood mortality and morbidity [2,3,4,5]. PTD is also a risk factor for long-term maternal health complications, including cardiovascular and renal morbidities [6,7].

In most countries, PTD rates are increasing, and an annual estimation of 15 million babies, which equates to 11.1% of live births, are born prematurely worldwide [8].

Spontaneous preterm parturition is a result of the pathological activation of one or more of the processes leading to delivery. The etiology of spontaneous preterm labor, which accounts for 70–83% of PTDs, is mostly unknown [9,10], and multiple etiologies are usually involved; these include cervical insufficiency, uterine ischemia, intrauterine infections, prior cervical surgeries, a decline in progesterone action (a critical hormone in pregnancy maintenance), uterine malformations, and others. Iatrogenic PTD may be indicated in cases of possible hazard to the mother or the fetus, mainly due to preeclampsia, fetal distress, or severe intrauterine growth restriction [11]. In addition to the differentiation between spontaneous or indicated, PTD can be categorized based on gestational age at delivery as follows: extreme preterm (<28 gestational weeks), very preterm (28–<32 gestational weeks), moderate preterm (32–<34 gestational weeks), and late preterm (34–<37 complete gestational weeks), the latter of which is when most PTDs occur [12].

Prevention strategies have been suggested and practiced to lower the risk of PTD; however, their effectiveness is questioned, especially among women considered to be at low risk for PTD [13,14,15]. These strategies may include progesterone administration, cervical cerclage, or antibiotic treatment.

Risk factors for PTD are obstetrical, socio-economical, behavioral, environmental, and genetic, and include young or old maternal age, infertility treatments, and smoking [16,17]. For instance, in this Special Issue, Kluwgant et al. mentions the high risk of extreme PTD among women with a history of placental abruption, placenta previa, and multiple gestation. Studies have shown variations in the effect size of the different risk factors based on the PTD type (spontaneous or indicated) and whether the case is initial PTD or recurrent PTD [18,19]. Studies addressing risk factors for PTD by type of PTD are presented in this Special Issue [16,18]. The leading risk factor for PTD is having a history of PTDs [19,20]; moreover, the risk increases with each additional PTD, or if the PTD occurred in the immediately preceding pregnancy, and if the gestational age of the first PTD was earlier [21]. The risk of recurrent spontaneous PTD varies among studies, from 18–31.6% [22,23].

One of the early detectable PTD risk factors is cervical shortening or insufficiency (cervical length ≤ 25 mm), defined as an inability of the cervix to remain closed during pregnancy [24]. However, cervical length was found to have a low predictive value, as only a small fraction of women with spontaneous preterm birth had a short cervix, and only half of those with a short cervix delivered prematurely [25]. Recurrent PTD, however, is more strongly associated with cervical length in the subsequent pregnancy, and it has a higher predictive value in recurrent compared to initial PTD [24,25].

Since PTD events are likely to re-occur in the same mother and within the family [26], factors that are persistent, such as genetic factors, or factors which are present over a long time span, such as exposure to environmental factors, are likely involved in PTD re-occurrence. Indeed, exposure to environmental factors such as smoking and air pollution have been associated with an increased risk of recurrent PTD [27,28]. Genetic studies based on whole-exome sequencing suggested an association between PTD and inflammatory regulation genes [29], as well as genes related to estrogen receptor alteration [30]. Although genetic factors have been associated with PTD risk, this has not been studied in association with recurrent PTD risk.

Fetal development occurs throughout the entire pregnancy until full term; therefore, when PTD occurs, the newborn is not physiologically and metabolically mature, leading to immediate and long-term complications [2,3,4,5]. The severity of these complications depends mainly on gestational age at delivery, and increases with reduced gestational age; this is reported by Gutvirtz et al. [3] and Zer at al. [4] in our Special Issue, in their works regarding risk of prematurity and respiratory or neurologic morbidities in offspring. In a large retrospective cohort, Gutvirtz et al. [3] found that offspring born at an extremely premature gestational age were ~3 times more likely to be hospitalized with respiratory morbidities, and Zer at al. [4] found that they were ~3.9 times more likely to be hospitalized with neurologic morbidities, compared to offspring born at term. Besides immediate and long-term health effects, PTD entails great economic consequences due to health costs, and the support of families and their offspring throughout life; this begins with the immediate hospitalization and intensive care treatment upon delivery. Aspects of the special treatment of premature newborns are also presented in this Special Issue by Saldana-Garcia et al. [31], who address the benefits of early interventions in reducing respiratory-related risk to the newborn. In the United States, the annual costs associated with PTD in the year 2005 were USD 26.2 billion [32].

Lowering the rate of this major, relatively prevalent pregnancy complication has been declared by the WHO as “an urgent priority for reaching Millennium Development Goal 4, calling for the reduction of child deaths” [33]. Although women with a history of PTD are clearly a population at risk for a subsequent PTD, and although strategies to prevent preterm birth have been practiced for over 30 years, the expectations have not been met, and PTD rates have not declined. Some PTD risk factors are preventable, and addressing them at the personal (e.g., patient education regarding prenatal product consumption and smoking) and population levels (e.g., industries and legislation), may decrease PTD incidence. Environmental policy has the potential to reduce PTD; this has recently been shown in the US, where legislation and health concerns have led to the closure of coal and oil power plants, and to the lowering of PTD incidence [34]. Even a small reduction in PTD incidence may have a large public-health and economic impact, in terms of preventing perinatal mortality, morbidity, and lifelong disability among affected infants.

We would like to give special thanks to the colleagues who took part in this project for their valuable contributions. 
